# Bioelectrical impedance analysis-derived phase angle at admission as a predictor of 90-day mortality in intensive care patients

**DOI:** 10.1038/s41430-018-0167-1

**Published:** 2018-05-11

**Authors:** Sandra N. Stapel, Wilhelmus G. P. M. Looijaard, Ingeborg M. Dekker, Armand R. J. Girbes, Peter J. M. Weijs, Heleen M Oudemans-van Straaten

**Affiliations:** 1 Department of Adult Intensive Care Medicine, VU University Medical Center, De Boelelaan 1117, Amsterdam, 1181 HV The Netherlands; 2 Research VUmc Intensive Care (REVIVE), VU University Medical Center, De Boelelaan 1117, Amsterdam, 1181 HV The Netherlands; 3 Institute of Cardiovascular Research (ICaR-VU), VU University Medical Center, De Boelelaan 1117, Amsterdam, 1181 HV The Netherlands; 4 Department of Internal Medicine, Nutrition and Dietetics, VU University Medical Center, De Boelelaan 1117, Amsterdam, 1181 HV The Netherlands

**Keywords:** Biomarkers, Risk factors

## Abstract

**Background/Objectives:**

A low bioelectrical impedance analysis (BIA)-derived phase angle (PA) predicts morbidity and mortality in different patient groups. An association between PA and long-term mortality in ICU patients has not been demonstrated before. The purpose of the present study was to determine whether PA on ICU admission independently predicts 90-day mortality.

**Subjects/ methods:**

This prospective observational study was performed in a mixed university ICU. BIA was performed in 196 patients within 24 h of ICU admission. To test the independent association between PA and 90-day mortality, logistic regression analysis was performed using the APACHE IV predicted mortality as confounder. The optimal cutoff value of PA for mortality prediction was determined by ROC curve analysis. Using this cutoff value, patients were categorized into low or normal PA group and the association with 90-day mortality was tested again.

**Results:**

The PA of survivors was higher than of the non-survivors (5.0° ± 1.3° vs. 4.1° ± 1.2°, *p* < 0.001). The area under the ROC curve of PA for 90-day mortality was 0.70 (CI 0.59–0.80). PA was associated with 90-day mortality (OR = 0.56, CI: 0.38–0.77, *p* = 0.001) on univariate logistic regression analysis and also after adjusting for BMI, gender, age, and APACHE IV on multivariable logistic regression (OR = 0.65, CI: 0.44–0.96, *p* = 0.031). A PA < 4.8° was an independent predictor of 90-day mortality (adjusted OR = 3.65, CI: 1.34–9.93, *p* = 0.011).

**Conclusions:**

Phase angle at ICU admission is an independent predictor of 90-day mortality. This biological marker can aid in long-term mortality risk assessment of critically ill patients.

## Introduction

Early identification of critically ill patients with a high risk of mortality is important to guide preventive and supportive measures. Several scoring systems have been developed to assess severity of acute and underlying disease and to predict mortality. Among them, the Acute Physiology and Chronic Health Evaluation (APACHE) IV system is the most recent and best validated international system and is used in the national intensive care databases for quality control [1]. The present scoring systems are designed to predict hospital mortality but not mortality beyond hospital discharge and can only be assessed 24 h after intensive care unit (ICU) admission. Furthermore, their calculation is cumbersome.

Bioelectrical impedance analysis (BIA)-derived phase angle (PA) is an alternative method to assess mortality risk. BIA is a simple, non-invasive technique that estimates body composition by measuring the opposition (impedance) to an applied current while passing through the body. Impedance consists of two components: resistance (R), which is the opposition to the flow of an alternating current through intra- and extracellular ionic solutions, and reactance (Xc), which is the delay in conduction as a result of capacitance by cell membranes and tissue interfaces. Capacitance causes a phase shift or PA that is quantified as arc tangent (Xc/*R*)*180°/*π* [2]. PA is regarded as a biological marker of cellular health, as it reflects cell mass, membrane integrity, and hydration status. PA has repeatedly proven to be a predictor of morbidity and mortality in various patient groups [3, 4]. Recent studies in critically ill patients showed that a low PA was associated with higher 28-day and hospital mortality risk [5–8]. An association between PA and long-term mortality risk in ICU patients has not been demonstrated before.

We hypothesize that ICU patients with a low PA at ICU admission have limited physiological reserve and will have a higher late mortality, often shortly after hospital discharge.

The aim of the present study was to determine whether PA on admission predicted 90-day mortality, independent of age, gender, body mass index (BMI), and APACHE IV predicted mortality rate.

## Materials and methods

### Setting and patients

This prospective observational study was performed over a 9-month period in the mixed medical/surgical ICU of the VU University Medical Centre. All adult patients admitted to the ICU were eligible for inclusion. Exclusion criteria were prostheses in extremities on both sides, limb amputation, implanted pacemaker, inability to lay still or supine, or skin defects on preferred electrode placement sites. Patients were included only when the primary investigator was present, which was mainly during weekdays and daytime hours (convenience sample). The institutional review board of the VU University Medical Center approved the study protocol and waived the need for written informed consent because of the low burden and risk associated with the study and the use of coded patient data.

### Bioelectrical impedance analysis

BIA was performed within 24 h of ICU admission with a BIA 101 Anniversary edition device (GLNP Life Sciences, AKERN, Florence, Italy). This single-frequency, phase-sensitive BIA device uses an alternating current of 400 μA with a frequency of 50 kHz. The patient is placed in supine position, with the extremities in a relaxed position not touching the body. Two pairs of electrodes were placed (source and sensor electrodes), one pair on the dorsum of the hand and one pair on the dorsum of the ipsilateral foot, with each electrode at 5 cm distance. After the application of an insensible current, the results for the resistance (ohm), reactance (ohm), and PA (degrees) are directly displayed on the BIA device and transferred to a database. As hydration status at the time of BIA-measurement influences PA, the hydration status of patients was classified using bioelectrical impedance vector analysis (BIVA). Patients were classified as having normal hydration, mild over- or underhydration, or severe over- or underhydration by comparing BIVA results with reference values derived from the normal population [9, 10].

### Other measurements

Demographic and clinical data, including gender, age, weight, height, BMI, APACHE II, III, and IV scores, admission diagnosis, and ICU-, hospital-, and 90-day mortality rates, were obtained from the patient data management system (PDMS; Metavision®; IMD-soft, Tel-Aviv, Israel) and the hospital information system (Mirador®; iSOFT Nederland BV, Leiden, The Netherlands). The Municipal Records Database was consulted as reference to determine if patients were still alive at 90 days after ICU admission.

### Statistics

Variables are expressed as number (%), mean (SD) or median (interquartile range) where appropriate. To compare results between survivors and non-survivors, the independent samples *T*-test, Mann–Whitney *U*-test, Fisher’s exact test, or *χ*^2^-test was used where appropriate.

To determine whether PA independently predicted 90-day mortality, univariate and multivariable logistic regression analyses were performed. Age, gender, BMI, and APACHE IV-predicted mortality were tested for confounding. Confounders were added as variable in the multivariable logistic regression analyses. To determine the discriminative capacity of PA for 90-day mortality, the receiver operator characteristics (ROC) curve was created and the area under the curve (AUC) calculated. To obtain a clinically relevant cutoff value, the PA yielding the highest sensitivity and specificity for 90-day mortality prediction was determined from the ROC curve (Youden’s index). Subsequently, patients were categorized into either the low or normal PA group according to this cutoff value and these groups were entered as categories in the multivariable logistic regression analysis. Finally, Kaplan–Meier curves for 90-day survival were constructed for the low and normal PA groups. Differences between curves were evaluated by a log-rank test.

We used SPSS IBM 22 (SPSS Inc., Chicago, IL, USA) for statistical analysis. A *p*-value of < 0.05 was considered statistically significant.

### Sample size calculation

Sample size was based on the ability to test the independent association between PA and 90-day mortality in a multivariate logistic regression model. Using a “validated rule of thumb” for logistic regression sample size calculations, with *k* = 3 independent covariates and an estimated 90-day mortality rate of 20%, a minimum of 150 patients were needed to attain sufficient power [11]. To account for loss to follow-up and the possibility of lower actual mortality, we aimed to recruit 200 patients.

## Results

During the study period, 1350 patients were admitted to the ICU with a mean age of 62.6 ± 16.1 years and a mean APACHE IV-predicted mortality score of 0.17 ± 0.21. BIA was performed in 202 patients within 24 h of ICU admission. Six patients were excluded from analysis because of erroneous values, most likely caused by muscle contractions during the measurement. Therefore, the results of 196 patients were analysed.

The median time interval between ICU admission and BIA measurement was 11 (8–13) h. Patient characteristics of all patients, and of 90-day survivors and non-survivors separately, are presented in Table 1. Mean BIA results for all, and of 90-day survivors and non-survivors separately, are presented in Table 2. The mean age of all patients was 64.8 ± 13.9 years and 67% of patients were male. Most patients were admitted after major surgery (56.1%). Mean PA was 4.9° ± 1.3°. In total, 30 patients died within 90 days of ICU admission (15.3%). Non-survivors had higher APACHE IV mortality prediction scores than survivors (0.55 ± 0.34 vs. 0.15 ± 0.22, *p* < 0.001).

**Table 1:** Patient characteristics of all patients, and of 90-day survivors and non-survivors separately

Characteristics	All patients (*n* = 196)	Survivors (*n* = 166)	Non-survivors (*n* = 30)	*p*-value
Age (years)	64.8 ± 13.9	64.3 ± 13.7	67.4 ± 14.7	0.267
Male gender (%)	131 (67)	115 (69)	16 (53)	0.088
Height (cm)	174 ± 9.5	173 ± 9.4	174 ± 10.5	0.773
Weight (kg)	80.0 ± 17.5	80.6 ± 17.8	80.0 ± 15.2	0.302
BMI (kg/m^2^)	26.4 ± 4.6	26.6 ± 4.7	25.5 ± 4.0	0.252
SOFA (1^st^ 24 h)	7.5 ± 2.9	7.1 ± 2.6	9.8 ± 3.3	< 0.001
APACHE II	22.9 ± 7.9	21.1 ± 7.1	28.0 ± 7.8	< 0.001
APACHE IV predicted mortality (fraction)	0.21 ± 0.28	0.15 ± 0.22	0.55 ± 0.34	< 0.001
Admission diagnosis (no., %)				
Cardiovascular	10 (5.1)	6 (3.6)	4 (13.3)	< 0.001
Metabolic/renal	7 (3.6)	5 (3.0)	2 (6.7)	0.322
Neurologic	5 (2.6)	5 (3.0)	0 (0)	0.337
Post resuscitation	22 (11.2)	13 (7.8)	9 (30)	< 0.001
Post major surgery	112 (57.1)	98 (59.0)	14 (46.7)	0.208
Respiratory insufficiency	13 (6.6)	9 (5.4)	4 (13.3)	0.109
Sepsis	12 (6.1)	11 (6.6)	1 (3.3)	0.490
Trauma	12 (6.1)	9 (5.4)	3 (10)	0.337
Others	3 (1.5)	2 (1.2)	1 (3.3)	0.384
Mechanical ventilation, (no., %)	170 (86.7)	145 (87.3)	25 (83.3)	0.549
BIVA hydration status, (no., %)				
Severe dehydration	0	0	0	
Mild dehydration	0	0	0	
Normal hydration	114 (58.2)	99 (59.6)	15 (50.0)	0.328
Mild overhydration	57 (29.1)	49 (29.5)	8 (26.7)	0.096
Severe overhydration	25 (12.8)	18 (10.8)	7 (23.3)	0.059

Values in mean ± SD or number (percentage). *APACHE* acute physiology and chronic health evaluation; *BIVA* bioelectrical impedance vector analysis; *BMI* body mass index; *ICU* intensive care unit; *SOFA* sequential organ failure assessment. *p*-values depict the statistical difference between 90-day survivors and non-survivors: independent *T*-test, equal variances not assumed

**Table 2:** Mean bioelectrical impedance analysis (BIA) results for all patients, and for 90-day survivors and non-survivors separately

Bioimpedance analysis results	All patients (*n* = 196)	Survivors (*n* = 166)	Non-survivors (*n* = 30)	*p*-value
Resistance (Ω)	459 ± 92	455 ± 89	476 ± 108	0.26
Reactance (Ω)	39.0 ± 11.3	39.8 ± 10.9	34.5 ± 12.7	0.02
Phase angle (°)^a^	4.9 ± 1.3	5.0 ± 1.3	4.1 ± 1.2	< 0.001

*p*-values depict the statistical difference between 90-day survivors and non-survivors: independent *T*-test, equal variances not assumed. ^a^ Phase angle = arc tangent of (Xc/*R*)*180°/*π*

### PA and 90-day mortality

The PA of 90-day survivors was significantly higher than of the non-survivors (5.0° ± 1.3° vs. 4.1° ± 1.2°, *p* < 0.001). Univariate logistic regression analysis showed that PA (as a continuous variable) and APACHE IV were associated with 90-day mortality (PA: odds ratio (OR) = 0.54, confidence interval (CI): 0.38–0.77, *p* = 0.001; APACHE IV: OR = 1.04, CI: 1.03–1.06, *p* < 0.001). BMI, gender, and age were not associated with 90-day mortality, nor were they confounders for the effect of PA on 90-day mortality. The association between PA and 90-day mortality remained significant when PA was adjusted for APACHE IV in the multivariable logistic regression analysis (adjusted OR = 0.65, CI: 0.44–0.96, *p* = 0.031). APACHE IV score also was an independent predictor of 90-day mortality (OR = 1.04, CI: 1.03–1.06, *p* < 0.001), see Table 3

**Table 3:** Univariable and multivariable logistic regression analysis for 90-day mortality

	Univariable		Multivariable phase angle, continuous		Multivariable phase angle, categorial	
Variable	Unadjusted OR (95% CI)	*p*-value	Adjusted OR^a^ (95% CI)	*p*-value	Adjusted OR^b^ (95% CI)	*p*-value
Phase angle (°)	0.54 (0.38–0.77**)**	0.001	0.65 (0.44–0.96)	0.031		
Low phase angle (< 4.8°)	3.77 (1.59–8.97)	0.003			3.65 (1.34–9.93)	0.011
APACHE IV predicted mortality	1.04 (1.03–1.06)	< 0.001	1.04 (1.03–1.06)	< 0.001	1.04 (1.03–1.06)	< 0.001
Age	1.02 (0.99–1.05)	0.267				
Gender	1.97 (0.90–4.34)	0.092				
BMI	0.95	0.251				

^a^Model summary: ^−2^logLH: 119.2, Nagelkerke *R*^2^: 0.381, Hosmer–Lemeshow test: *χ*^2^: 8.971, *p* = 0.345

^b^Model summary: ^−2^logLH: 117.33, Nagelkerke *R*^2^: 0.395, Hosmer–Lemeshow test: *χ*^2^: 9.021, *p* = 0.341

Univariable and multivariable logistic regression analysis showing the unadjusted and adjusted odds ratios for 90-day mortality. ^a^ Phase angle entered as a continuous variable. ^b^ Phase angle entered as a categorical variable (with a cutoff of 4.8°)

Age, gender, BMI, and APACHE IV-predicted mortality were tested for confounding. Age, gender, and BMI were no confounders. APACHE IV-predicted mortality was a confounder and was adjusted for in the multivariable logistic regression analysis

Abbreviations: APACHE IV-predicted mortality, derived from the acute physiology and chronic health evaluation system expressed as fraction (range 0–1); *BMI* body mass index; *CI* confidence interval; *OR* odds ratio

**Fig. 1:**
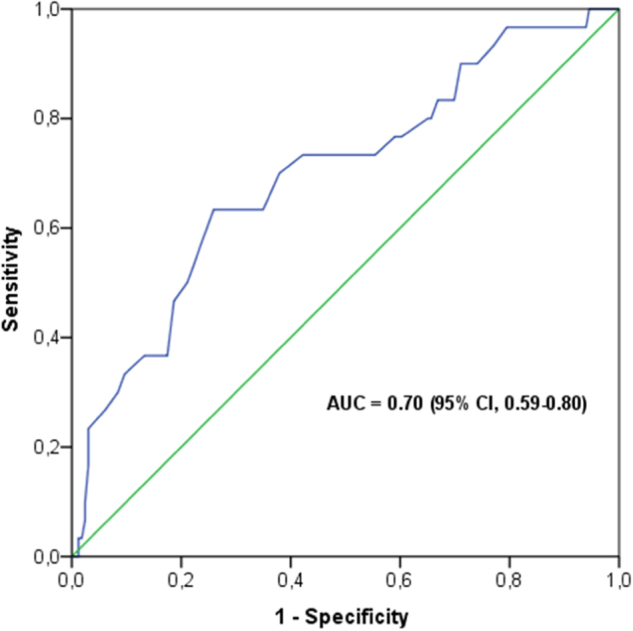
The receiver operating characteristics (ROC) curve of phase angle for 90-day mortality. The area under the curve (AUC) is 0.70 (95% CI = 0.59–0.80)

The AUC of the ROC curve of PA for 90-day mortality was 0.70 (CI: 0.59–0.80), see Fig. 1.

### Optimal PA cutoff value and 90-day mortality prediction

**Fig. 2:**
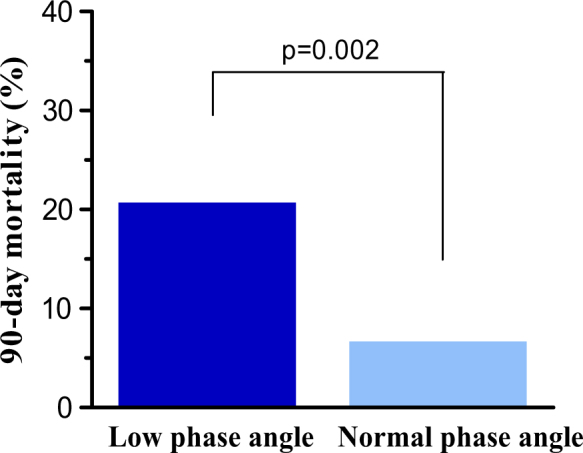
90-Day mortality in patients with low ( < 4.8°) or normal phase angle ( ≥ 4.8°)

**Fig. 3:**
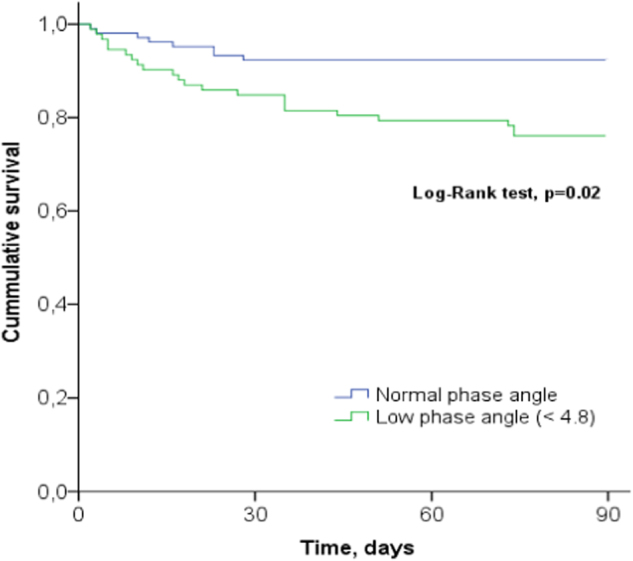
Kaplan–Meier 90-days survival plot illustrating cumulative survival for patients with a low phase angle ( < 4.8°) or normal phase angle ( ≥ 4.8°). Log-rank test, *p* = 0.02

The optimal PA cutoff value, derived from the ROC-curve, was 4.8°, yielding a sensitivity of 0.73 and a specificity of 0.58. The 90-day mortality rate was significantly higher in patients with low PA than in patients with normal PA (23.9 vs. 7.7%, *p* < 0.002, Fig. 2). A low PA was a predictor for 90-day mortality in the univariate logistic regression analysis (OR = 3.77, CI: 1.59–8.97, *p* = 0.029) and remained an independent predictor in the multivariable logistic regression analysis after adjusting for APACHE IV score (adjusted OR = 3.65, CI: 1.34–9.93, *p* = 0.011) (Table 3). APACHE IV score also was an independent predictor of 90-day mortality (OR = 1.04, CI: 1.03–1.06, *p* < 0.001), see Table 3. Figure 3 shows the 90-day survival as Kaplan–Meier curves for the low vs. normal PA group. Mortality in the low PA group continued to increase during the 90 day study period, whereas mortality of the patients with a PA above 4.8° did not increase after 28 days (see Fig. 3).

## Discussion

This prospective observational study in ICU patients shows that BIA-derived PA at ICU admission predicted 90-day mortality. Patients with a PA below 4.8° had a 3.7 times higher adjusted risk of dying. These findings are in line with other studies reporting the prognostic value of PA for clinical outcome. The present study is the first study reporting the relation between the PA at ICU admission and long-term (90-day) mortality.

PA, being a function of both resistance and reactance, reflects the proportion of cellular mass, the integrity of cell membranes and hydration status, and represents a biological marker of cellular health [2]. PA declines with age and sarcopenia [12, 13], and a low PA is associated with malnutrition and frailty [4, 14, 15]. PA may therefore reflect limited physiological reserve, which explains its association with long-term mortality. A low PA on ICU admission is influenced by both the acute illness, as a result of membrane dysfunction and fluid shifts, and by the underlying general condition. The measurement of PA is easy, non-invasive, cheap, of low risk, and not restricted to the intensive care setting, and is therefore an attractive biological marker that is applicable for long-term follow up outside the intensive care setting.

Most previous studies in critically ill patients demonstrated the prognostic value of PA for short-term mortality. In a study in 95 critically ill patients, da Silva et al. [5] found that PA was a good prognostic marker for patients without sepsis, but not for the septic cohort. In a multicenter study by Kuchnia et al. [6] including 71 critically ill patients, low PA predicted time to live ICU discharge, but only when PA cutoffs derived from the National Health and Nutrition Examination Survey were used, not with PA as continuous value. In the subsequent large international multicenter observational landmark study, day-1 PA was independently associated with 28-day mortality [7]. Remarkably, Lee et al. [8] found that PA had stronger hospital mortality predictive power than several disease severity scores used in the ICU. In two small, single-center studies performed in Brazil, the mortality rate in the low PA group was higher, but there was no significant association between PA and mortality [16, 17]. The mean PA differed among the above mentioned studies (4.0° to 5.4°) as well as the prognostic strength of PA to predict mortality, with unadjusted AUCs varying between 0.53 and 0.77. Differences can be explained by differences in design (single or multicenter), case mix (age, ethnicity, surgical or mixed surgical/medical admissions, severity of disease, mortality), the type of mortality (hospital, 28-day, 90-day), the BIA device used, and the interval between ICU admission and PA assessment. The study of Thibault et al. [7] points to the importance of the timing of BIA measurement: although day-1 PA predicted mortality, day-5 PA did not. A recent study of Kuchnia et al. [18] showed that ethnicity is an important variable that should be accounted for when determining population reference values for PA. Finally, the present study considered long-term mortality, thereby including the late mortality risk of patients with a low PA at ICU admission. The association between PA and long-term mortality has been demonstrated in cancer patients by Norman et al. [19]. They showed that in cancer patients older than 60 years, a PA below the fifth reference percentile was predictive of decreased muscle strength, impaired quality of life, and 1-year mortality [19]. Our results suggest that PA has even stronger discriminative power when used for prognostication beyond 28-day mortality. The survival curves in our population showed a substantial late mortality in patients with a low PA, underscoring the potential of PA as a predictor of late mortality.

The question remains whether PA is a reliable marker of cellular health and muscularity during all phases of critical illness. Previously, we have shown that a low muscle mass, as measured by computed tomography scanning on ICU admission, is an independent predictor of hospital mortality and of discharge to a nursing home [20]. Muscle mass is an important marker for both risk stratification and outcome. Kuchnia et al. [6] highlighted the potential use of PA as a marker to identify patients with low muscularity. BIA-derived PA is considered as a surrogate for fat-free mass [7]. Sarcopenic patients have lower PA values, whereas the PA of athletes is high [12, 13]. Future studies are necessary to investigate if PA is a valid surrogate for fat-free mass, especially in critically ill patients with altered hydration status. PA is an attractive index, because it is independent of body weight but, being a function of resistance and reactance, BIA also changes with altering hydration status. Therefore, large fluid shifts before ICU admission or during the first hours of an ICU stay could cause changes in the BIA-derived PA, which likely reflect inflammation-induced changes in membrane integrity causing fluid redistribution into the extracellular space. In that case, low PA not only reflects body cell mass but also the consequences of altered hydration status [4]. The influence of altered hydration on PA may explain why day-5 PA (in contrast with day-1 PA) was not discriminative for mortality in the study by Thibault et al. [7]. Measuring PA early after admission will likely reduce the confounding of altered hydration. BIVA was used to assess hydration status of the studied patients [9, 10]. Non-surviving patients were more often “overhydrated” and “severely overhydrated” according to BIVA. However, the difference was not significant, but might become so if sample size would be larger.

Interestingly, no patients were classified as dehydrated by BIVA. The median time to BIA measurement in our study was 11 h (8–13). It is possible that fluid resuscitation before or in the first hours of ICU admission led to “normal- or overhydration” at time of measurement. Patients in the “severe overhydration” group likely have a higher degree of inflammation and subsequently decreased cell membranes integrity with fluid shift from intracellular to extracellular, leading to lower PA values. In contrast to other studies, we did not find a correlation between PA and APACHE IV or SOFA (sequential organ failure assessment) scores [5, 7]. Reason may be that low PA not only reflects acute changes but also poor underlying health, muscle wasting, and fragility, which are poorly reflected by the APACHE II score.

Our study has several limitations. We used a convenience sample, meaning BIA measurements were only performed when the researcher was present, thus introducing selection bias by including less acute admissions during off hours. However, baseline characteristics and disease severity scores of the studied patients were equal to those of all patients admitted during the study period and comparable to other studies [5–8, 16, 17]. Another limitation is that the optimal PA cutoff value of 4.8° was derived from the studied patients and not independently (internal validity). However, our cutoff value for PA is equal to the cutoff of 4.8° found in patients with heart failure [21] and corresponds to the cutoff of 5° found in cancer patients to predict functionality, quality of life, and mortality [22]. Of note, our PA cutoff value of 4.8° is lower than the PA derived from a large data set of more than 210,000 healthy Germans. In this data set, the mean PA of gender and BMI-matched healthy individuals was 6.01° ± 0.75° for males and 5.59° ± 0.72° for females, with tenth percentile values of 5.14 and 4.79, respectively [23]. In a smaller data set of 1967 healthy subject from the United States, the PA of age-matched individuals was 6.96° ± 1.10° for males and 5.97° ± 0.83° for females [24]. Cutoff values are population specific as shown by the differences between published studies. Furthermore, the sensitivity of our cutoff value was reasonable, but specificity was poor, suggesting that a low PA identifies the patients at risk of dying reasonably well, but a considerable number of patients with a low PA will survive up to 90 days after ICU admission. Using cutoff values facilitates implementation of PA measurements in clinical practice; however, ideally, the cutoff value used should be prospectively validated in a large cohort of ICU patients.

In conclusion, the present study shows that BIA-derived PA at ICU admission is an independent predictor of 90-day mortality. PA is a biological marker that can aid in long-term mortality risk assessment and may be used to monitor targeted interventions aiming to improve long-term outcome of ICU patients. Future studies should aim at investigating the confounding effect of altered hydration on PA measurement during the course of ICU admission and whether interventions aiming to improve long-term functional status, such as increasing protein intake and early mobilization, also increase PA. In that case, PA is an even more attractive monitoring tool.
